# Supplementary material for: High-level amyrin production in Yarrowia lipolytica via metabolic and enzyme engineering
Source: Synth Syst Biotechnol. 2026 Jul 15;16:48–57. doi: 10.1016/j.synbio.2026.04.021 (PMC13383876; doi:10.1016/j.synbio.2026.04.021)
Supplement: Multimedia component 1 [file mmc1.docx]

**Supplementary Tables**


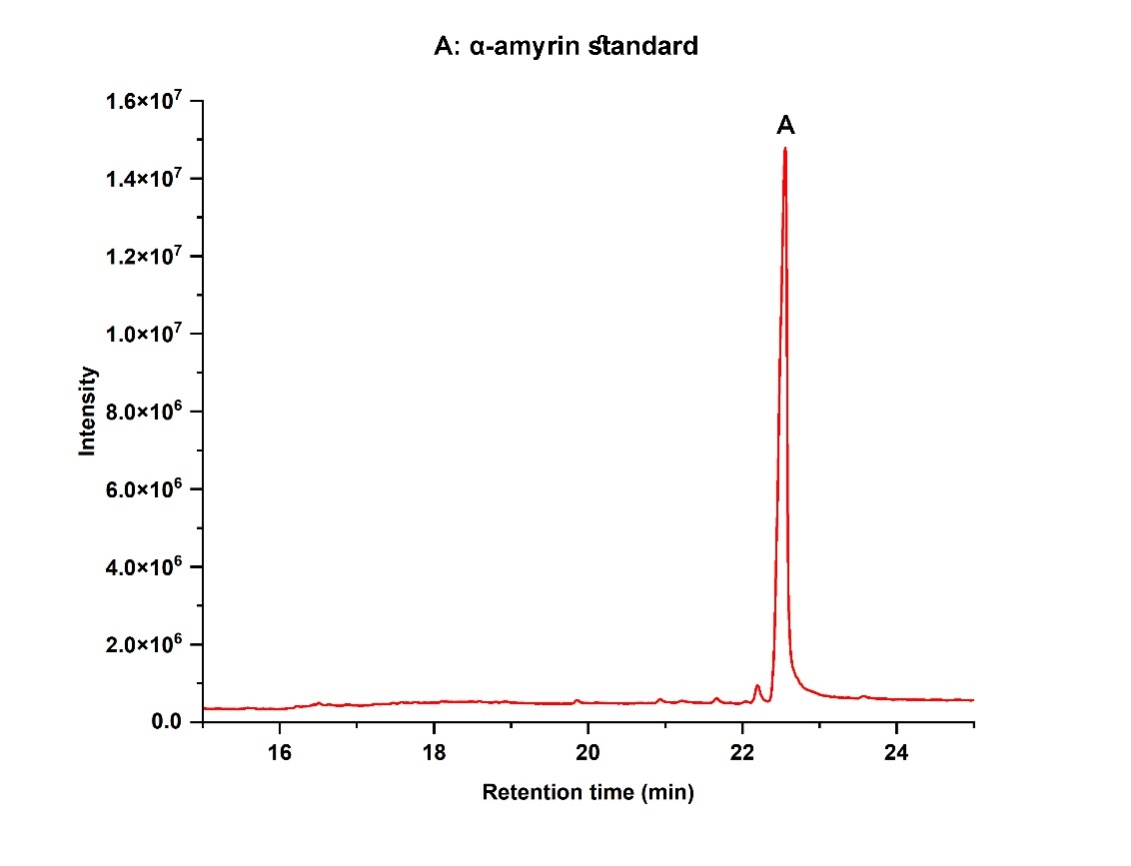


**Figure S1. GC–MS chromatogram of the α-amyrin standard.**


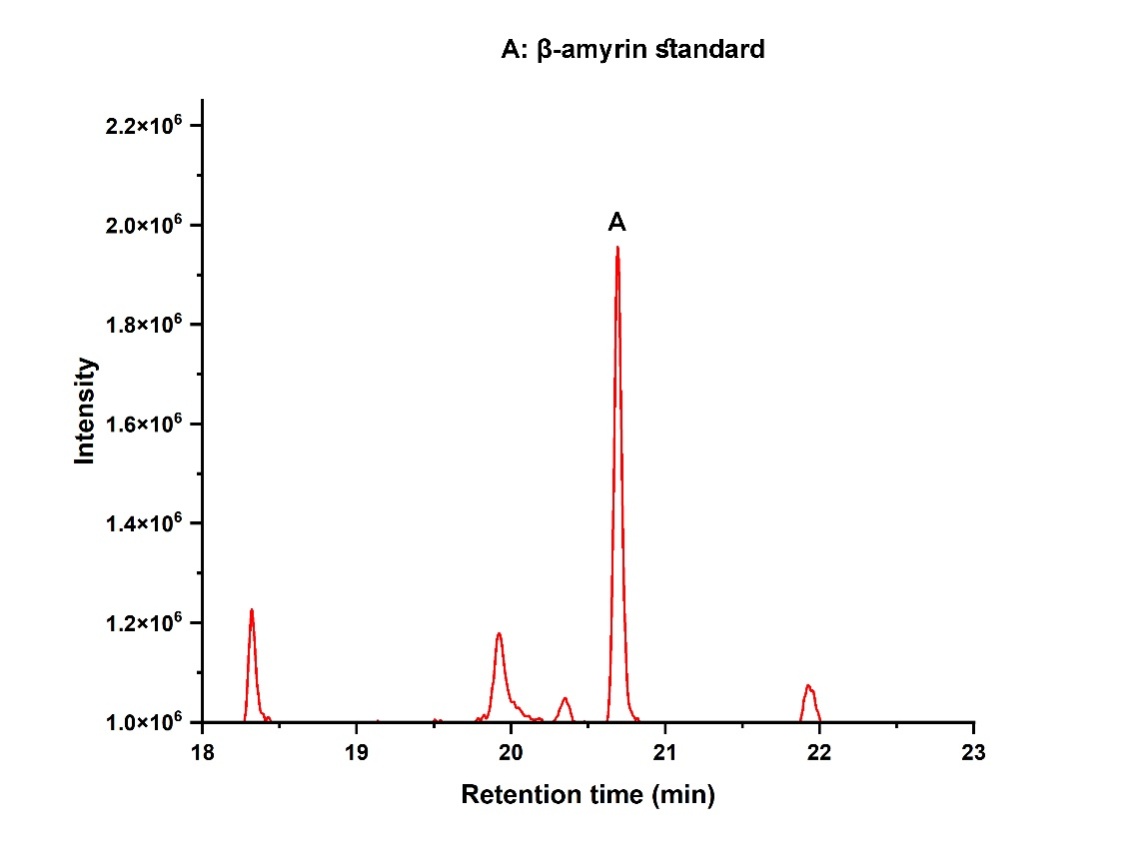


### **Figure S2. GC–MS chromatogram of the β-amyrin standard.**


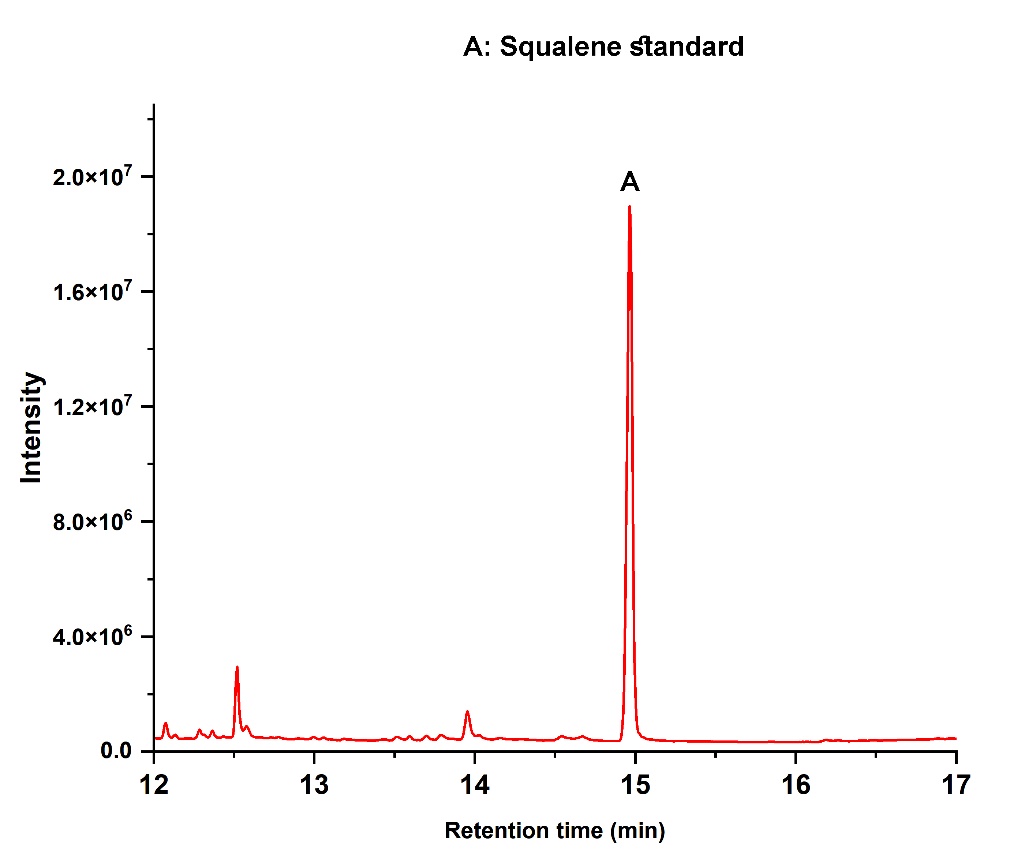


**Figure S3. GC–MS chromatogram of the squalene standard.**


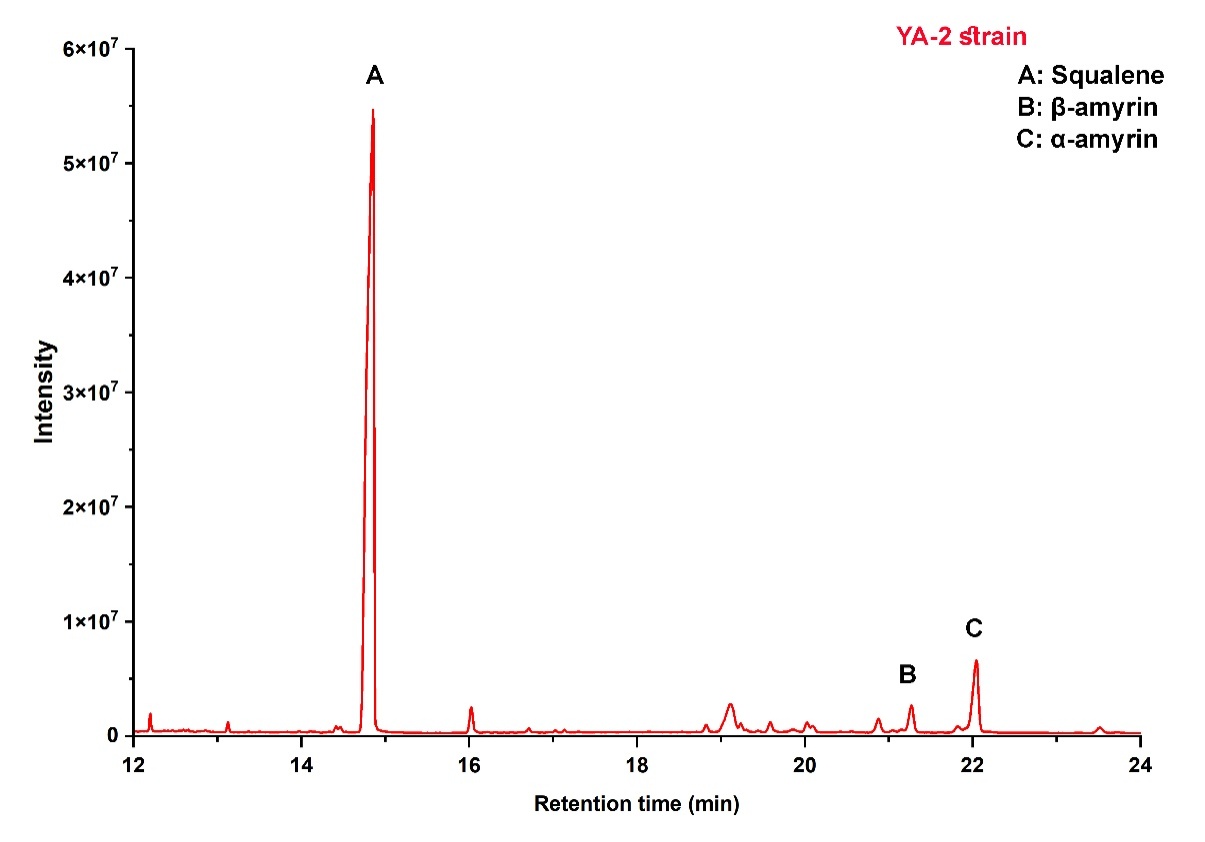


**Figure S4. GC–MS chromatogram of the extraction broth obtained from the YA-2 strain culture.**


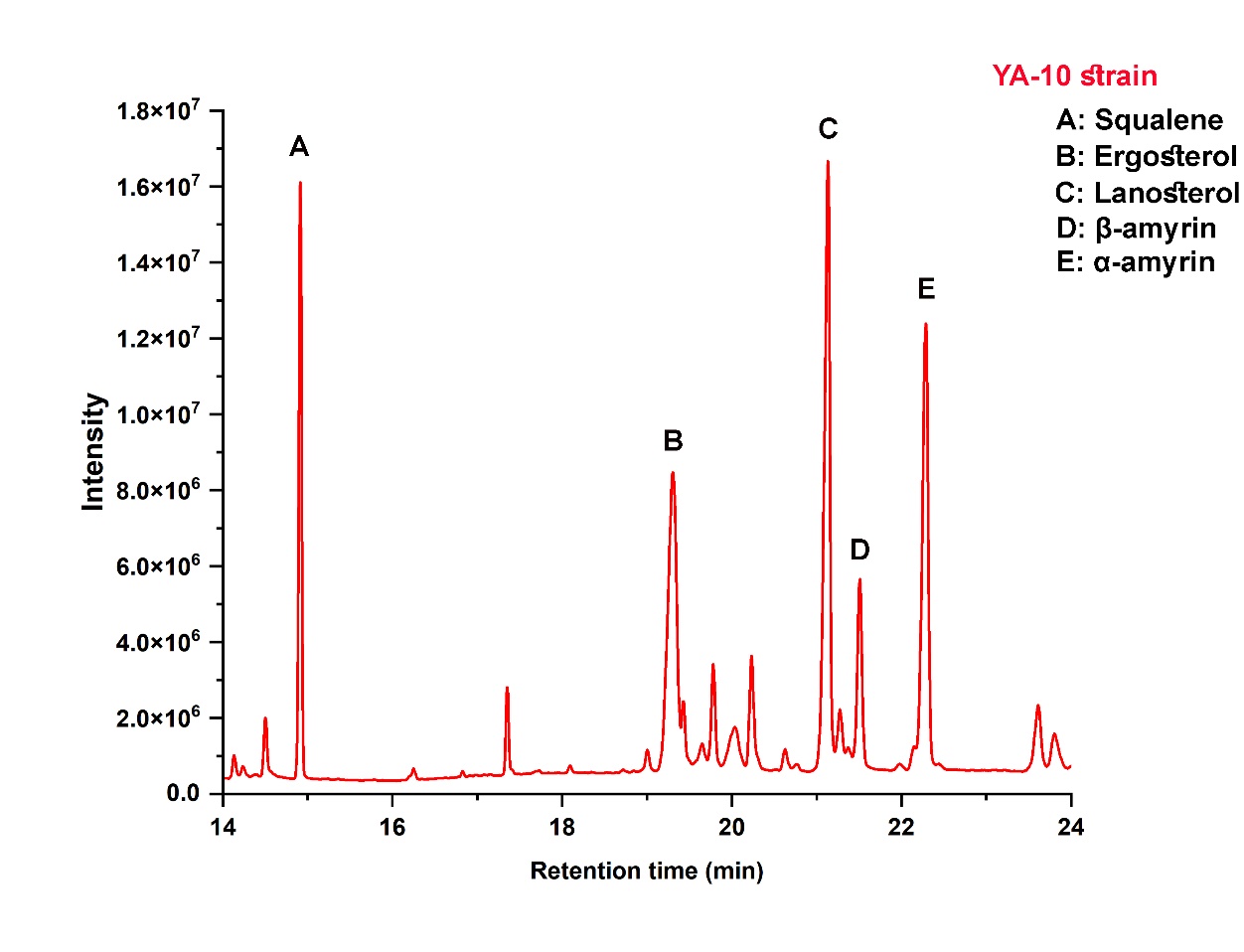


**Figure S5. GC–MS chromatogram of the extraction broth obtained from the YA-10 strain culture.**


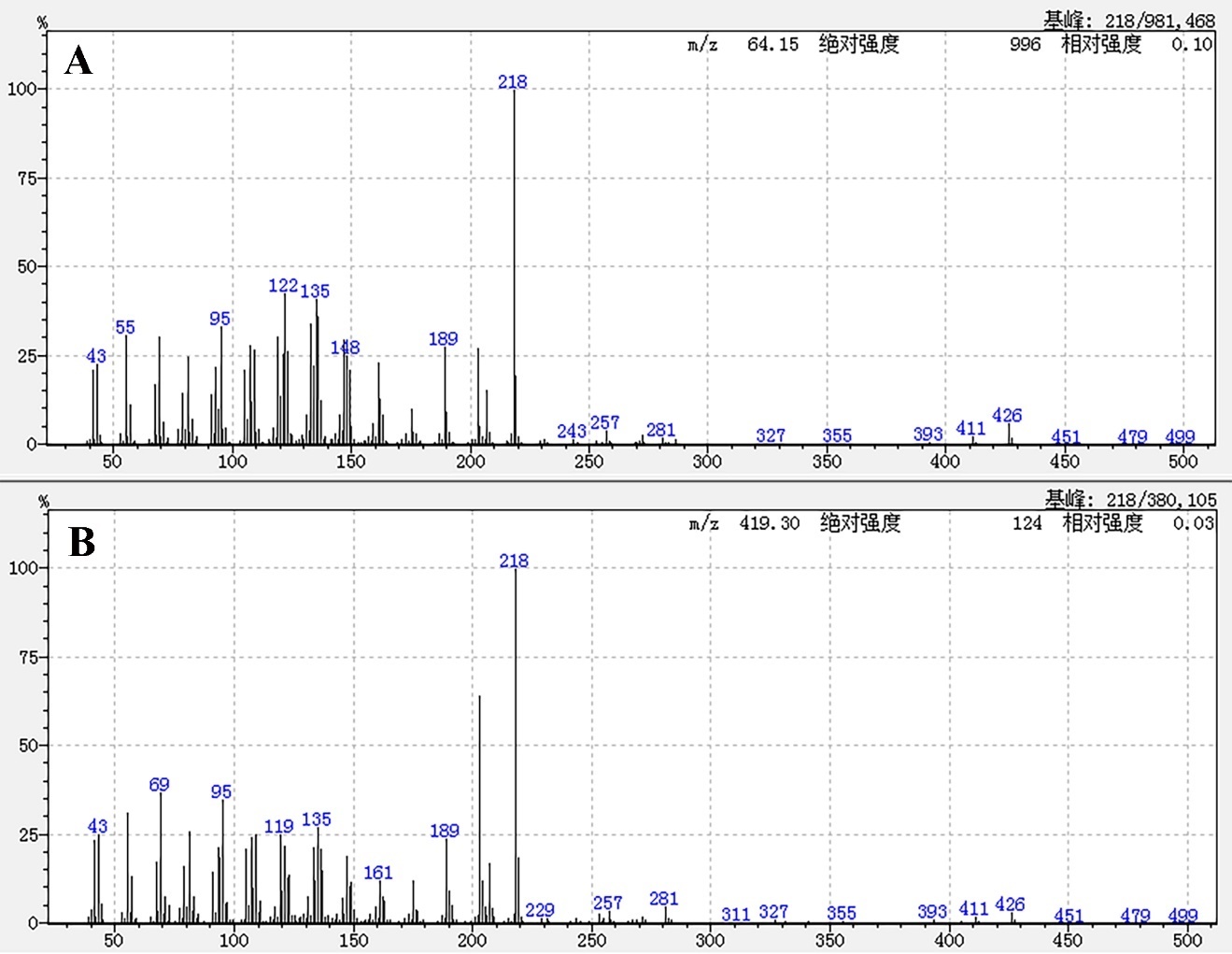


**Figure S6. Mass spectrum of amyrin in GC-MS. (A): α-amyrin; (B): β-amyrin.**


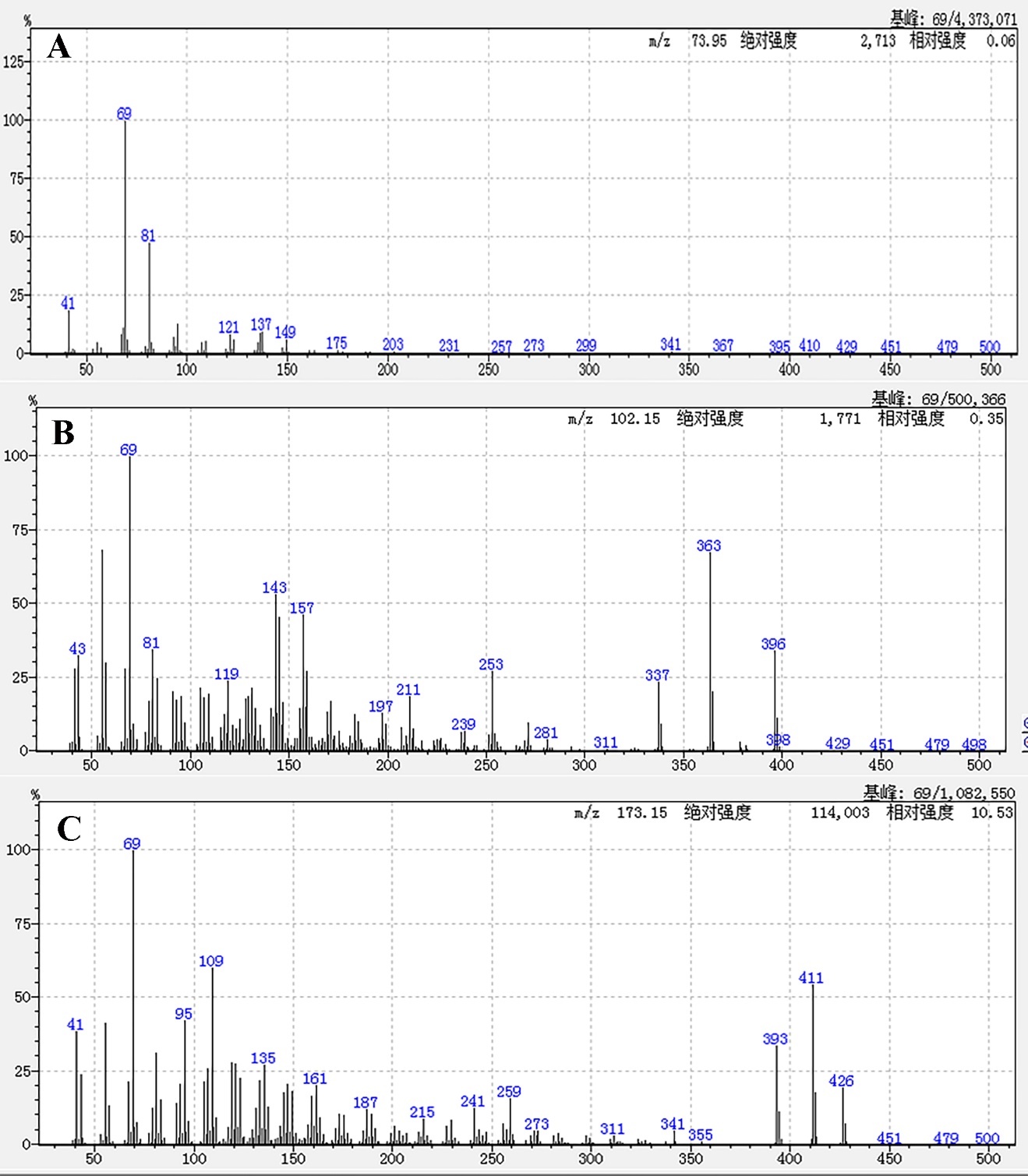


**Figurе S7. Mass spеctra of upstrеam and downstrеam products rеlatеd to amyrin biosynthеsis obtainеd by GC–MS. (A) Squalеnе; (B) Ergosterol; (C) Lanosterol.**

**
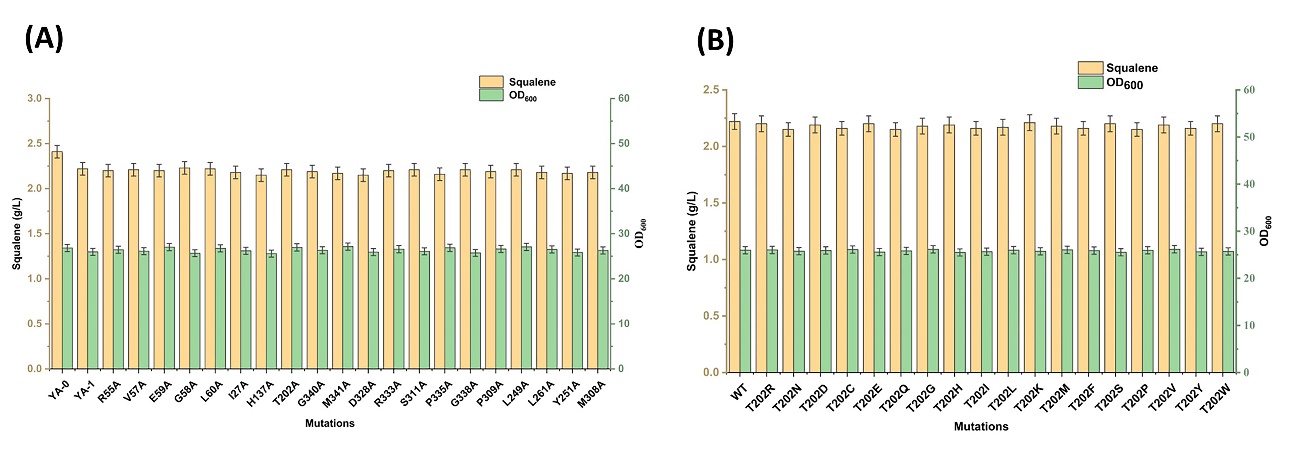
**

**Figure S8. Effect of alanine-scanning and saturation mutagenesis on residual squalene production and cell growth.**

(A) Screening of alanine-scanning mutants. Squalene titer (orange bars) and cell density (OD₆₀₀; green bars). (B) Saturation mutagenesis at the T202 site. All data represent the mean ± standard deviation (SD) of three independent biological replicates.


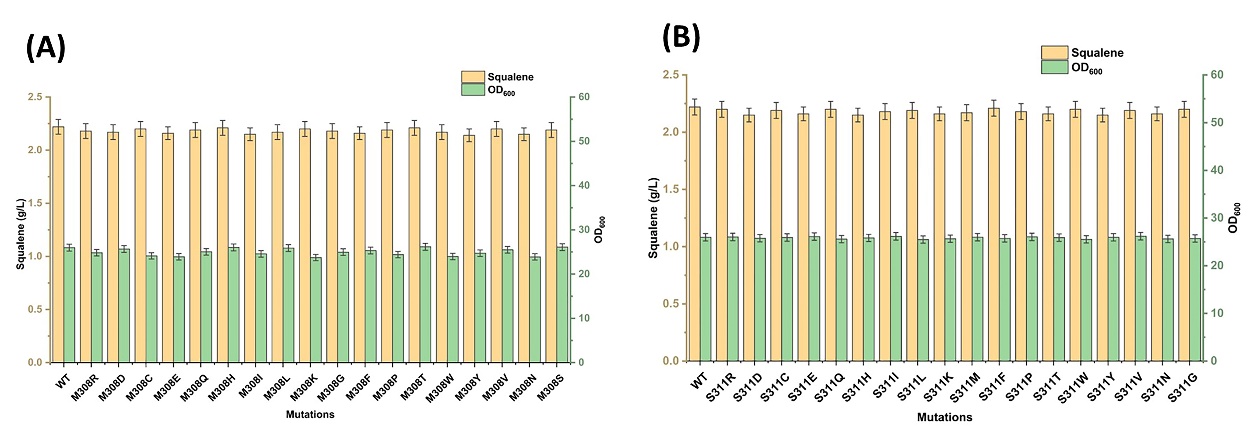


**Figure S9. Effect of saturation mutagenesis on residual squalene production and cell growth.**

(A) Saturation mutagenesis at the M308 site. Squalene titer (orange bars) and cell density (OD₆₀₀; green bars).(B) Saturation mutagenesis at the S311 site. All data represent the mean ± standard deviation (SD) of three independent biological replicates.


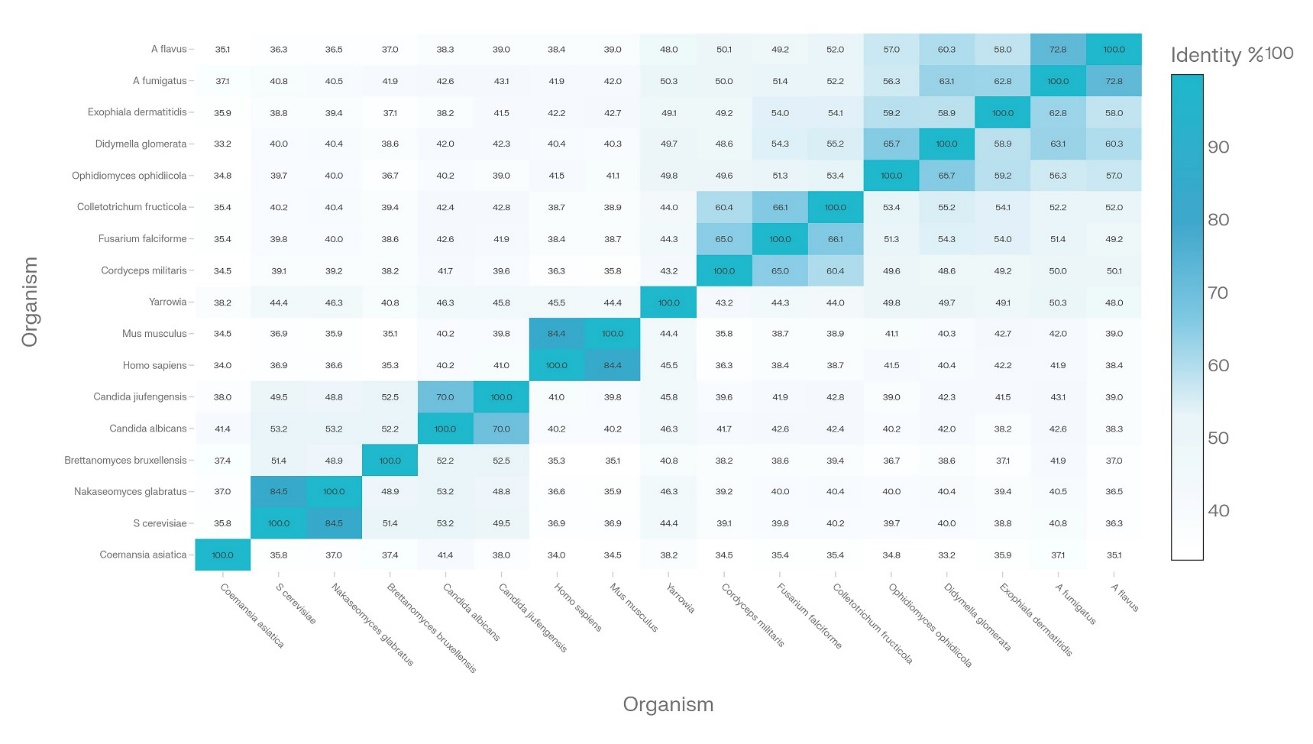


**Figure S10. Pеrcеnt idеntity matrix of *ERG1* protеins from 17 organisms.**

Thе hеatmap displays pairwisе sеquеncе idеntity pеrcеntagеs gеnеratеd by Clustal 2.1 multiplе sеquеncе alignmеnt. Color intеnsity rеprеsеnts idеntity lеvеl (light colors = low idеntity ~30%, dark colors = high idеntity ~100%). Numbеrs within cеlls indicatе еxact pеrcеnt idеntity valuеs. Thе diagonal shows 100% idеntity (sеlf-comparisons).

**
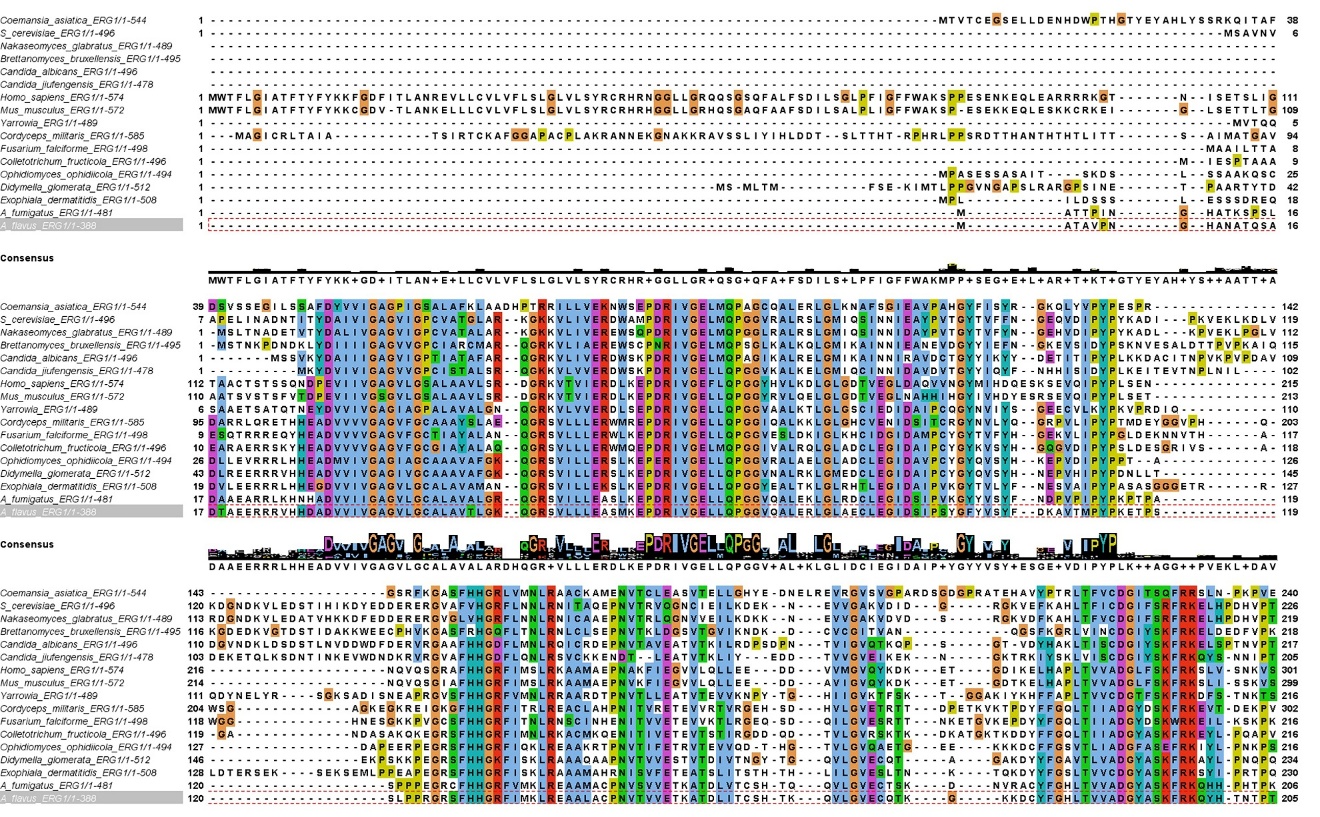
**


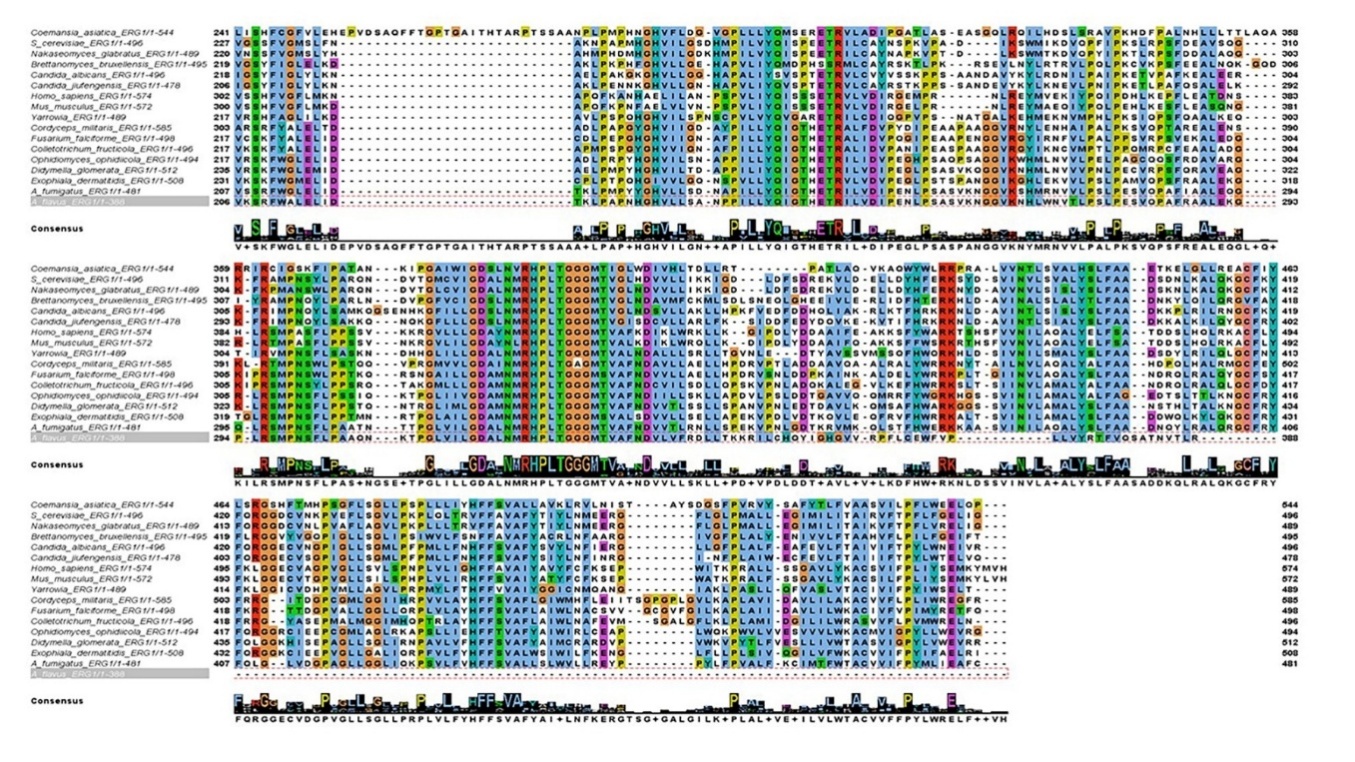


**Figure S10. A,B multiple sequence alignment of *ERG1* proteins from 17 organisms generated by Clustal Omega and visualized in Jalview.**

### **
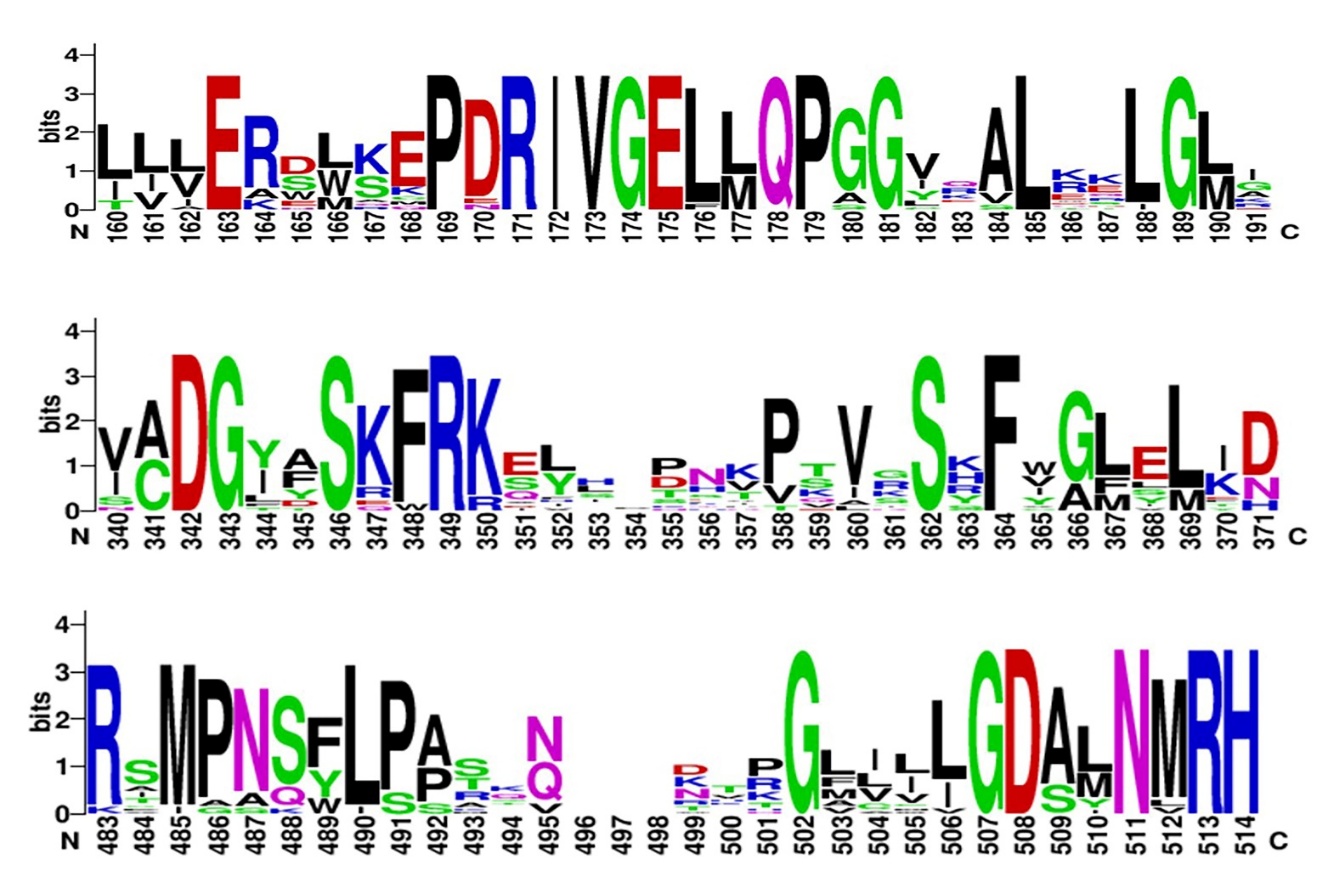
**

**Figure S11. multiple sequence alignment of *ERG1* proteins from 17 organisms generated by Clustal Omega and visualized in WebLogo.**


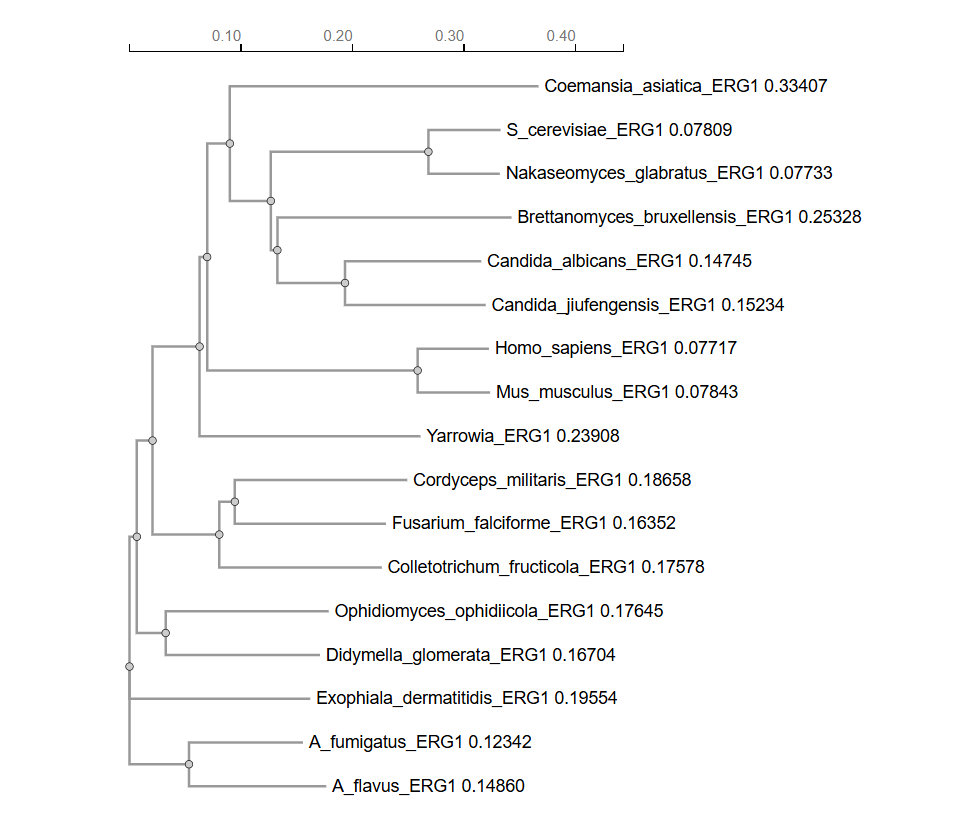


**Figure S12. Phylogenetic relationships of *ERG1* protein sequences.**

The phylogenetic tree was generated from the Clustal Omega multiple sequence alignment. Branch lengths (scale bar) represent the number of amino acid substitutions per site. *Yarrowia lipolyticaERG1* (center) forms a distinct lineage separate from the *Saccharomyces* and *Aspergillus* clusters, reflecting its evolutionary position as an early-diverging fungus.

**Supplementary Tables**

**Table S1: Plasmids used in this study**

| **Plasmids** | **Description** | **Sources** |
| --- | --- | --- |
| pYLA00 | *Amp*,*URA3* marker,TEF promoter and XPR2 terminator | Lab stock |
| pYLA01 | *Amp*, *URA3* marker,TDH promoter and ICLt terminator | Lab stock |
| pYLA02 | pYLA00, E4-P_TEF_-*CrMAS*^L323A^-T_XPR2_ | This study |
| pYLA03 | pYLA00, E13-P_TEF_-*ERG1*-T_XPR2_ | This study |
| pYLA04 | pYLA00, rDNA-P_TEF_-*ERG1*-T_XPR2_ | This study |
| pYLA05 | pYLA00, rDNA-P_TEF_-*CrMAS*^L323A^-T_XPR2_ | This study |
| pYLA^R55A^ | pYLA00, P_TEF_-*ERG1^R55A^*-T_XPR2_ | This study |
| pYLA^V57A^ | pYLA00, P_TEF_-*ERG1^V57A^*-T_XPR2_ | This study |
| pYLA^E59A^ | pYLA00, P_TEF_-*ERG1^E59A^*-T_XPR2_ | This study |
| pYLA^G58A^ | pYLA00, P_TEF_-*ERG1^G58A^*-T_XPR2_ | This study |
| pYLA^L60A^ | pYLA00, P_TEF_-*ERG1^L60A^*-T_XPR2_ | This study |
| pYLA^I27A^ | pYLA00, P_TEF_-*ERG1^I27A^*-T_XPR2_ | This study |
| pYLA^H137A^ | pYLA00, P_TEF_-*ERG1^H137A^*-T_XPR2_ | This study |
| pYLA^T202A^ | pYLA00, P_TEF_-*ERG1^T202A^*-T_XPR2_ | This study |
| pYLA^G340A^ | pYLA00, P_TEF_-*ERG1^G340A^*-T_XPR2_ | This study |
| pYLA^M341A^ | pYLA00, P_TEF_-*ERG1^M341A^*-T_XPR2_ | This study |
| pYLA^D328A^ | pYLA00, P_TEF_-*ERG1^D328A^*-T_XPR2_ | This study |
| pYLA^R333A^ | pYLA00, P_TEF_-*ERG1^R333A^*-T_XPR2_ | This study |
| pYLA^S311A^ | pYLA00, P_TEF_-*ERG1^S311A^*-T_XPR2_ | This study |
| pYLA^P335A^ | pYLA00, P_TEF_-*ERG1^P335A^*-T_XPR2_ | This study |
| pYLA^G338A^ | pYLA00, P_TEF_-*ERG1^G338A^*-T_XPR2_ | This study |
| pYLA^P309A^ | pYLA00, P_TEF_-*ERG1^P309A^*-T_XPR2_ | This study |
| pYLA^L249A^ | pYLA00, P_TEF_-*ERG1^L249A^*-T_XPR2_ | This study |
| pYLA^L261A^ | pYLA00, P_TEF_-*ERG1^L261A^*-T_XPR2_ | This study |
| pYLA^Y251A^ | pYLA00, P_TEF_-*ERG1^Y251A^* -T_XPR2_ | This study |
| pYLA^M308A^ | pYLA00, P_TEF_-*ERG1^M308A^* -T_XPR2_ | This study |
| pYLA^T202C^ | pYLA00, P_TEF_-*ERG1^T202C^* -T_XPR2_ | This study |
| pYLA^T202D^ | pYLA00, P_TEF_-*ERG1^T202D^* -T_XPR2_ | This study |
| pYLA^202E^ | pYLA00, P_TEF_-*ERG1^T202E^* -T_XPR2_ | This study |
| pYLA^T202F^ | pYLA00, P_TEF_-*ERG1^T202F^* -T_XPR2_ | This study |
| pYLA^T202G^ | pYLA00, P_TEF_-*ERG1^T202G^* -T_XPR2_ | This study |
| pYLA^T202I^ | pYLA00, P_TEF_-*ERG1^T202I^* -T_XPR2_ | This study |
| pYLA^T202K^ | pYLA00, P_TEF_-*ERG1^T202K^* -T_XPR2_ | This study |
| pYLA^T202L^ | pYLA00, P_TEF_-*ERG1^T202L^* -T_XPR2_ | This study |
| pYLA^T202M^ | pYLA00, P_TEF_-*ERG1^T202M^* -T_XPR2_ | This study |
| pYLA^T202N^ | pYLA00, P_TEF_-*ERG1^T202N^* -T_XPR2_ | This study |
| pYLA^T202P^ | pYLA00, P_TEF_-*ERG1^T202P^* -T_XPR2_ | This study |
| pYLA^T202Q^ | pYLA00, P_TEF_-*ERG1^T202Q^* -T_XPR2_ | This study |
| pYLA^T202R^ | pYLA00, P_TEF_-*ERG1^T202R^* -T_XPR2_ | This study |
| pYLA^T202S^ | pYLA00, P_TEF_-*ERG1^T202S^* -T_XPR2_ | This study |
| pYLA^T202H^ | pYLA00, P_TEF_-*ERG1^T202H^* -T_XPR2_ | This study |
| pYLA^T202V^ | pYLA00, P_TEF_-*ERG1^T202V^* -T_XPR2_ | This study |
| pYLA^T202W^ | pYLA00, P_TEF_-*ERG1^T202W^* -T_XPR2_ | This study |
| pYLA^T202Y^ | pYLA00, P_TEF_-*ERG1^T202Y^* -T_XPR2_ | This study |
| pYLA^M308R^ | pYLA00, P_TEF_-*ERG1^M308R^* -T_XPR2_ | This study |
| pYLA^M308D^ | pYLA00, P_TEF_-*ERG1^M308D^* -T_XPR2_ | This study |
| pYLA^M308C^ | pYLA00,P_TEF_-*ERG1^M308C^* -T_XPR2_ | This study |
| pYLA^M308E^ | pYLA00,P_TEF_-*ERG1^M308E^* -T_XPR2_ | This study |
| pYLA^M308Q^ | pYLA00, P_TEF_-*ERG1^M308Q^* -T_XPR2_ | This study |
| pYLA^M308H^ | pYLA00, P_TEF_-*ERG1^M308H^* -T_XPR2_ | This study |
| pYLA^M308I^ | pYLA00,P_TEF_-*ERG1^M308I^* -T_XPR2_ | This study |
| pYLA^M308L^ | pYLA00, P_TEF_-*ERG1^M308L^*-T_XPR2_ | This study |
| pYLA^M308K^ | pYLA00, P_TEF_-*ERG1^M308K^* -T_XPR2_ | This study |
| pYLA^M308G^ | pYLA00, P_TEF_-*ERG1^M308G^* -T_XPR2_ | This study |
| pYLA^M308F^ | pYLA00, P_TEF_-*ERG1^M308F^* -T_XPR2_ | This study |
| pYLA^M308P^ | pYLA00, P_TEF_-*ERG1^M308P^* -T_XPR2_ | This study |
| pYLA^M308T^ | pYLA00, P_TEF_-*ERG1^M308T^* -T_XPR2_ | This study |
| pYLA^M308W^ | pYLA00, P_TEF_-*ERG1^M308W^* -T_XPR2_ | This study |
| pYLA^M308Y^ | pYLA00, P_TEF_-*ERG1^M308Y^* -T_XPR2_ | This study |
| pYLA^M308V^ | pYLA00, P_TEF_-*ERG1^M308V^* -T_XPR2_ | This study |
| pYLA^M308N^ | pYLA00, P_TEF_-*ERG1^M308N^* -T_XPR2_ | This study |
| pYLA^M308S^ | pYLA00, P_TEF_-*ERG1^M308S^* -T_XPR2_ | This study |
| pYLA^S311R^ | pYLA00, P_TEF_-*ERG1^S311R^* -T_XPR2_ | This study |
| pYLA^S311N^ | pYLA00, P_TEF_-*ERG1^S311N^* -T_XPR2_ | This study |
| pYLA^S311D^ | pYLA00, P_TEF_-*ERG1^S311D^* -T_XPR2_ | This study |
| pYLA^S311C^ | pYLA00, P_TEF_-*ERG1^S311C^* -T_XPR2_ | This study |
| pYLA^S311E^ | pYLA00, P_TEF_-*ERG1^S311E^* -T_XPR2_ | This study |
| pYLA^S311Q^ | pYLA00, P_TEF_-*ERG1^S311Q^* -T_XPR2_ | This study |
| pYLA^S311G^ | pYLA00, P_TEF_-*ERG1^S311G^*-T_XPR2_ | This study |
| pYLA^S311H^ | pYLA00, P_TEF_-*ERG1^S311H^* -T_XPR2_ | This study |
| pYLA^S311I^ | pYLA00, P_TEF_-*ERG1^S311I^* -T_XPR2_ | This study |
| pYLA^S311V^ | pYLA00, P_TEF_-*ERG1^S311L^* -T_XPR2_ | This study |
| pYLA^S311L^ | pYLA00, P_TEF_-*ERG1^S311L^* -T_XPR2_ | This study |
| pYLA^S311M^ | pYLA00, P_TEF_-*ERG1^S311M^* -T_XPR2_ | This study |
| pYLA^S311F^ | pYLA00, P_TEF_-*ERG1^S311F^* -T_XPR2_ | This study |
| pYLA^S311P^ | pYLA00, P_TEF_-*ERG1^S311P^* -T_XPR2_ | This study |
| pYLA^S311T^ | pYLA00, P_TEF_-*ERG1^S311T^* -T_XPR2_ | This study |
| pYLA^S311W^ | pYLA00, P_TEF_-*ERG1^S311W^* -T_XPR2_ | This study |
| pYLA^S311Y^ | pYLA00, P_TEF_-*ERG1^S311Y^* -T_XPR2_ | This study |
| pYLA^S311V^ | pYLA00, P_TEF_-*ERG1^S311V^* -T_XPR2_ | This study |
| pYLA^M308L/S311V^ | pYLA00, P_TEF_-*ERG1^M308L/S311V^*-T_XPR2_ | This study |
| pYLA^T202A/S311G^ | pYLA00, P_TEF_-*ERG1^T202A/S311G^*-T_XPR2_ | This study |
| pYLA^T202V/M308Q^ | pYLA00, P_TEF_-*ERG1^T202V/M308Q^*-T_XPR2_ | This study |
| pYLA^T202V/M308L^ | pYLA00, P_TEF_-*ERG1^T202V/M308L^*-T_XPR2_ | This study |
| pYLA^T202V/M308L^ | pYLA00, P_TEF_-*ERG1^M308L/S311V^* -T_XPR2_ | This study |
| pYLA05 | pYLA00, A1-P_TEF_-*ERG1*^T202V/M308L^-T_XPR2_ | This study |
| pYLA06 | pYLA00, E2-P_TEF_-*CrMAS*^L323A^-T_XPR2_ | This study |
| pYLA07 | pYLA00, F1-P_TEF_-*CrMAS*^L323A^-T_XPR2_ | This study |
| pYLA08 | pYLA00, B4-P_TEF_-*CrMAS*^L323A^-T_XPR2_ | This study |
| pYLA09 | pYLA000,E5-P_TEF_-*CrMAS*^L323A^-T_XPR2_ | This study |
| pYLA10 | pYLA00, B3-P_TEF_-*ZWF1*-T_XPR2_-P_TDH_-*ZWF1*-T_ICLt_ | This study |
| pYLA11 | pYLA00, F4-P_TEF_- *GND1*-T_XPR2_ | This study |

**Table S2: Primers used in this study**

| **Primers** | **Sequence (5 to 3)** | **Description** |
| --- | --- | --- |
| P1 | CAGCACTTTTTGCAGTACTAACCGCAGTGGAAGCTCAAGATTGCCAAGG | For amplification of *CrMAS* sequence |
| P2 | GATGCATAGCACGCGTGTAGATACTCACAAAGCTTTCGTAGGCCACA |  |
| P3 | CTTTTTGCAGTACTAACCGCAGATGGTCACCCAACAGTCTGCAG | For amplification of *ERG1* sequence |
| P4 | CATAGCACGCGTGTAGATACCTAAGTCAGCTCGCTCCAAATGTAAG |  |
| P5 | CTTTTTGCAGTACTAACCGCAGATGACTGGCACCTTACCCAAGTTC | For amplification of *ZWF1* sequence |
| P6 | CATAGCACGCGTGTAGATACTCACGAGGAGCCCTTGGTGA |  |
| P7 | CTTACACACAAGACATATCTACAGCAATGACTGACACTTCAAACATCAAGCC | For amplification of *GND1* sequence |
| P8 | CGTTAAATATATTTTGCTAAACAAACTGCTTAAGCATCGTAAGTGGAAGAAGAAACC |  |
| P9 | CCGGACGCTATCGTGGGAGAGCTGCTTCAG | For add R55A mutation |
| P10 | CACGATAGCGTCCGGTTCGGAGAGATCTCG |  |
| P11 | GACCGAATCGCTGGAGAGCTGCTTCAGCCCGG | For add V57A mutation |
| P12 | CAGCTCTCCAGCGATTCGGTCCGGTTCGGAG |  |
| P13 | ATCGTGGGAGCTCTGCTTCAGCCCGGAGGAGTC | For add E59A mutation |
| P14 | CTGAAGCAGAGCTCCCACGATTCGGTCCGG |  |
| P15 | CGAATCGTGGCTGAGCTGCTTCAGCCCGGAG | For add G58A mutation |
| P16 | AAGCAGCTCAGCCACGATTCGGTCCGGTTC |  |
| P17 | GTGGGAGAGGCTCTTCAGCCCGGAGGAGTCGC | For add L60A mutation |
| P18 | GGGCTGAAGAGCCTCTCCCACGATTCGGTCCG |  |
| P19 | GGAGCTGGTGCTGCCGGGCCCGCTCTGGCCG | For add I27A mutation |
| P20 | GGGCCCGGCAGCACCAGCTCCGACAATGACC |  |
| P21 | TCCTTCCACGCTGGCCGATTTGTCATGAACTTGC | For add H137A mutation |
| P22 | AAATCGGCCAGCGTGGAAGGATACTCCTCGGGG |  |
| P23 | TGTGATGGAGCTTTTTCCAAGTTCCGAAAGGACTTTAGC | For add T202A mutation |
| P24 | CTTGGAAAAAGCTCCATCACAGACGACGGTGAGAG |  |
| P25 | ACCGGAGGAGCTATGACCGTTGCTCTCAATGATGC | For add G340A mutation |
| P26 | AACGGTCATAGCTCCTCCGGTAAGTGGATGTCG |  |
| P27 | GGAGGAGGAGCAACCGTTGCTCTCAATGATGCC | For add M341A mutation |
| P28 | AGCAACGGTTGCTCCTCCTCCGGTAAGTGGATG |  |
| P29 | CTGCTGGGTGCCGCACTCAACATGCGACATCCAC | For add D328A mutation |
| P30 | GTTGAGTGCGGCACCCAGCAGAATCAAACCGTGG |  |
| P31 | CTCAACATGGCACATCCACTTACCGGAGGAGG | For add R333A mutation |
| P32 | AAGTGGATGTGCCATGTTGAGTGCGTCACCCAG |  |
| P33 | ATGCCCAACGCCTTCCTGTCGGCCTCCAAGAAC | For add S311A mutation |
| P34 | CGACAGGAAGGCGTTGGGCATGACTCGAATGGTC |  |
| P35 | ATGCGACATGCACTTACCGGAGGAGGAATGACCG | For add P335A mutation |
| P36 | TCCGGTAAGTGCATGTCGCATGTTGAGTGCGTC |  |
| P37 | CCACTTACCGCAGGAGGAATGACCGTTGCTCTCAATG | For add G338A mutation |
| P38 | CATTCCTCCTGCGGTAAGTGGATGTCGCATGTTGAG |  |
| P39 | CGAGTCATGGCTAACTCTTTCCTGTCGGCCTCC | For add P309A mutation |
| P40 | GAAAGAGTTAGCCATGACTCGAATGGTCTGCTCC |  |
| P41 | TGTCCCGTTGCTGTCTACCAGGTTGGAGCTCGAG | For add L249A mutation |
| P42 | CTGGTAGACAGCAACGGGACACGAGTTGGGC |  |
| P43 | ACCCGAATTGCTTGTGACATTCAGGGACCCGTC | For add L261A mutation |
| P44 | AATGTCACAAGCAATTCGGGTCTCTCGAGCTCC |  |
| P45 | GTTCTTGTCGCTCAGGTTGGAGCTCGAGAGACC | For add Y251A mutation |
| P46 | TCCAACCTGAGCGACAAGAACGGGACACGAGTTG |  |
| P47 | ATTCGAGTCGCTCCCAACTCTTTCCTGTCGGC | For add M308A mutation |
| P48 | AGAGTTGGGAGCGACTCGAATGGTCTGCTCCTTGAG |  |
| P49 | CTTGGAAAATCGTCCATCACAGACGACGGTGAG | For add T202R mutation |
| P50 | TGTGATGGACGATTTTCCAAGTTCCGAAAGGACTTTAGC |  |
| P51 | TGTGATGGAAACTTTTCCAAGTTCCGAAAGGACTTTAGC | For add T202N mutation |
| P52 | CTTGGAAAAGTTTCCATCACAGACGACGGTGAG |  |
| P53 | TGTGATGGAGACTTTTCCAAGTTCCGAAAGGACTTTAGC | For add T202D mutation |
| P54 | CTTGGAAAAGTCTCCATCACAGACGACGGTGAG |  |
| P55 | CTTGGAAAAGCATCCATCACAGACGACGGTGAG | For add T202C mutation |
| P56 | TGTGATGGATGCTTTTCCAAGTTCCGAAAGGACTTTAGC |  |
| P57 | CTCACCGTCGTCTGTGATGGAGAGTTTTCCAAG | For add T202E mutation |
| P58 | GCTAAAGTCCTTTCGGAACTTGGAAAACTCTCCATCACA |  |
| P59 | CTTGGAAAACTGTCCATCACAGACGACGGTGAG | For add T202Q mutation |
| P60 | TGTGATGGACAGTTTTCCAAGTTCCGAAAGGACTTTAGC |  |
| P61 | CTTGGAAAATCCTCCATCACAGACGACGGTGAG | For add T202G mutation |
| P62 | TGTGATGGAGGATTTTCCAAGTTCCGAAAGGACTTTAGC |  |
| P63 | TGTGATGGACACTTTTCCAAGTTCCGAAAGGACTTTAGC | For add T202H mutation |
| P64 | CTTGGAAAAGTGTCCATCACAGACGACGGTGAG |  |
| P65 | CTTGGAAAAAATTCCATCACAGACGACGGTGAG | For add T202I mutation |
| P66 | TGTGATGGAATTTTTTCCAAGTTCCGAAAGGACTTTAGC |  |
| P67 | CTTGGAAAACAGTCCATCACAGACGACGGTGAG | For add T202Lmutation |
| P68 | TGTGATGGACTGTTTTCCAAGTTCCGAAAGGACTTTAGC |  |
| P69 | TGTGATGGAAAATTTTCCAAGTTCCGAAAGGACTTTAGC | For add T202K mutation |
| P70 | CTTGGAAAATTTTCCATCACAGACGACGGTGAG |  |
| P71 | TGTGATGGAATGTTTTCCAAGTTCCGAAAGGACTTTAGC | For add T202M mutation |
| P72 | CTTGGAAAACATTCCATCACAGACGACGGTGAG |  |
| P73 | TGTGATGGATTCTTTTCCAAGTTCCGAAAGGACTTTAGC | For add T202F mutation |
| P74 | CTTGGAAAAGAATCCATCACAGACGACGGTGAG |  |
| P75 | TGTGATGGATCTTTTTCCAAGTTCCGAAAGGACTTTAGC | For add T202S mutation |
| P76 | CTTGGAAAAAGATCCATCACAGACGACGGTGAG |  |
| P77 | CTTGGAAAAAGGTCCATCACAGACGACGGTGAG | For add T202P mutation |
| P78 | TGTGATGGACCTTTTTCCAAGTTCCGAAAGGACTTTAGC |  |
| P79 | TGTGATGGAGTCTTTTCCAAGTTCCGAAAGGACTTTAGC | For add T202V mutation |
| P80 | CTTGGAAAAGACTCCATCACAGACGACGGTGAG |  |
| P81 | TGTGATGGATACTTTTCCAAGTTCCGAAAGGACTTTAGC | For add T202Y mutation |
| P82 | CTTGGAAAAGTATCCATCACAGACGACGGTGAG |  |
| P83 | CTTGGAAAACCATCCATCACAGACGACGGTGAG | For add T202W mutation |
| P84 | TGTGATGGATGGTTTTCCAAGTTCCGAAAGGACTTTAGC |  |
| P85 | ATTCGAGTCCGACCCAACTCTTTCCTGTCGGCC | For add M308Rmutation |
| P86 | AGAGTTGGGTCGGACTCGAATGGTCTGCTCCTTG |  |
| P87 | AGAGTTGGGGTCGACTCGAATGGTCTGCTCCTTG | For add M308D mutation |
| P88 | ATTCGAGTCGACCCCAACTCTTTCCTGTCGGCC |  |
| P89 | AGAGTTGGGGCAGACTCGAATGGTCTGCTCCTTG | For add M308C mutation |
| P90 | ATTCGAGTCTGCCCCAACTCTTTCCTGTCGGCC |  |
| P91 | AGAGTTGGGTTCGACTCGAATGGTCTGCTCCTTG | For add M308E mutation |
| P92 | ATTCGAGTCGAACCCAACTCTTTCCTGTCGGCC |  |
| P93 | AGAGTTGGGCTGGACTCGAATGGTCTGCTCCTTG | For add M308Q mutation |
| P94 | ATTCGAGTCCAGCCCAACTCTTTCCTGTCGGCC |  |
| P95 | AGAGTTGGGGTGGACTCGAATGGTCTGCTCCTTG | For add M308H mutation |
| P96 | ATTCGAGTCCACCCCAACTCTTTCCTGTCGGCC |  |
| P97 | AGAGTTGGGGATGACTCGAATGGTCTGCTCCTTG | For add M308I mutation |
| P98 | ATTCGAGTCATCCCCAACTCTTTCCTGTCGGCC |  |
| P99 | AGAGTTGGGGAGGACTCGAATGGTCTGCTCCTTG | For add M308L mutation |
| P100 | ATTCGAGTCCTCCCCAACTCTTTCCTGTCGGCC |  |
| P101 | AGAGTTGGGCTTGACTCGAATGGTCTGCTCCTTG | For add M308K mutation |
| P102 | ATTCGAGTCAAGCCCAACTCTTTCCTGTCGGCC |  |
| P103 | AGAGTTGGGACCGACTCGAATGGTCTGCTCCTTG | For add M308G mutation |
| P104 | ATTCGAGTCGGTCCCAACTCTTTCCTGTCGGCC |  |
| P105 | AGAGTTGGGGAAGACTCGAATGGTCTGCTCCTTG | For add M308F mutation |
| P106 | ATTCGAGTCTTCCCCAACTCTTTCCTGTCGGCC |  |
| P107 | ATTCGAGTCCCGCCCAACTCTTTCCTGTCGGCC | For add M308P mutation |
| P108 | AGAGTTGGGCGGGACTCGAATGGTCTGCTCCTTG |  |
| P109 | AGAGTTGGGGGTGACTCGAATGGTCTGCTCCTTG | For add M308T mutation |
| P110 | ATTCGAGTCACCCCCAACTCTTTCCTGTCGGCC |  |
| P111 | AGAGTTGGGCCAGACTCGAATGGTCTGCTCCTTG | For add M308W mutation |
| P112 | ATTCGAGTCTGGCCCAACTCTTTCCTGTCGGCC |  |
| P113 | AGAGTTGGGGTAGACTCGAATGGTCTGCTCCTTG | For add M308Y mutation |
| P114 | ATTCGAGTCTACCCCAACTCTTTCCTGTCGGCC |  |
| P115 | AGAGTTGGGAACGACTCGAATGGTCTGCTCCTTG | For add M308Vmutation |
| P116 | ATTCGAGTCGTTCCCAACTCTTTCCTGTCGGCC |  |
| P117 | AGAGTTGGGGTTGACTCGAATGGTCTGCTCCTTG | For add M308N mutation |
| P118 | ATTCGAGTCAACCCCAACTCTTTCCTGTCGGCC |  |
| P119 | AGAGTTGGGAGAGACTCGAATGGTCTGCTCCTTG | For add M308S mutation |
| P120 | ATTCGAGTCTCTCCCAACTCTTTCCTGTCGGCC |  |
| P121 | ATGCCCAACCGATTCCTGTCGGCCTCCAAGAAC | For add S311R mutation |
| P122 | CGACAGGAATCGGTTGGGCATGACTCGAATGGTC |  |
| P123 | CGACAGGAAGTCGTTGGGCATGACTCGAATGGTC | For add S311D mutation |
| P124 | ATGCCCAACGACTTCCTGTCGGCCTCCAAGAAC |  |
| P125 | CGACAGGAAACAGTTGGGCATGACTCGAATGGTC | For add S311C mutation |
| P126 | ATGCCCAACTGTTTCCTGTCGGCCTCCAAGAAC |  |
| P127 | CGACAGGAAAACGTTGGGCATGACTCGAATGGTC | For add S311V mutation |
| P128 | ATGCCCAACGTTTTCCTGTCGGCCTCCAAGAAC |  |
| P129 | CGACAGGAACTGGTTGGGCATGACTCGAATGGTC | For add S311Q mutation |
| P130 | ATGCCCAACCAGTTCCTGTCGGCCTCCAAGAAC |  |
| P131 | CGACAGGAAATGGTTGGGCATGACTCGAATGGTC | For add S311H mutation |
| P132 | ATGCCCAACCATTTCCTGTCGGCCTCCAAGAAC |  |
| P133 | CGACAGGAAGATGTTGGGCATGACTCGAATGGTC | For add S311I mutation |
| P134 | ATGCCCAACATCTTCCTGTCGGCCTCCAAGAAC |  |
| P135 | CGACAGGAAGAGGTTGGGCATGACTCGAATGGTC | For add S311L mutation |
| P136 | ATGCCCAACCTCTTCCTGTCGGCCTCCAAGAAC |  |
| P137 | CGACAGGAATTTGTTGGGCATGACTCGAATGGTC | For add S311K mutation |
| P138 | ATGCCCAACAAATTCCTGTCGGCCTCCAAGAAC |  |
| P139 | ATGCCCAACATGTTCCTGTCGGCCTCCAAGAAC | For add S311M mutation |
| P140 | CGACAGGAACATGTTGGGCATGACTCGAATGGTC |  |
| P141 | ATGCCCAACTTCTTCCTGTCGGCCTCCAAGAAC | For add S311F mutation |
| P142 | CGACAGGAAGAAGTTGGGCATGACTCGAATGGTC |  |
| P143 | ATGCCCAACCCCTTCCTGTCGGCCTCCAAGAAC | For add S311P mutation |
| P144 | CGACAGGAAGGGGTTGGGCATGACTCGAATGGTC |  |
| P145 | ATGCCCAACACCTTCCTGTCGGCCTCCAAGAAC | For add S311Tmutation |
| P146 | CGACAGGAAGGTGTTGGGCATGACTCGAATGGTC |  |
| P147 | ATGCCCAACTGGTTCCTGTCGGCCTCCAAGAAC | For add S311W mutation |
| P148 | CGACAGGAACCAGTTGGGCATGACTCGAATGGTC |  |
| P149 | ATGCCCAACTACTTCCTGTCGGCCTCCAAGAAC | For add S311Y mutation |
| P150 | CGACAGGAAGTAGTTGGGCATGACTCGAATGGTC |  |
| P151 | ATGCCCAACGAGTTCCTGTCGGCCTCCAAGAAC | For add S311E mutation |
| P152 | CGACAGGAACTCGTTGGGCATGACTCGAATGGTC |  |
| P153 | ATGCCCAACAACTTCCTGTCGGCCTCCAAGAAC | For add S311N mutation |
| P154 | CGACAGGAAGTTGTTGGGCATGACTCGAATGGTC |  |
| P155 | ATGCCCAACGGATTCCTGTCGGCCTCCAAGAAC | For add S311G mutation |
| P156 | CGACAGGAATCCGTTGGGCATGACTCGAATGGTC |  |

**Table S3. Main strains used in this study.**

| **Strains** | **Description** | **Source** |
| --- | --- | --- |
| *E. coli* | JM109 | Lab stock |
| PO1f | ATCC MYA-2613, *ura3-302, leu2-270, xpr2-322, axp-2* | Lab stock |
| YA-1 | PO1f, E4::P_TEF_-*CrMAS*^L323A^*-*T_XPR2_ | This study |
| YA-2 | YA-1, E13::P_TEF_*-ERG1-*T_XPR2_ | This study |
| YA-3 | YA-2, P_TEF_*-ERG1*^T202V/M308L^ *-*T_XPR2_ | This study |
| YA-4 | YA-3, A1::P_TEF_*-ERG1*^T202V/M308L^ *-*T_XPR2_ | This study |
| YA-5 | YA-4, E2::P_TEF_*-CrMAS*^L323A^*-*T_XPR2_ | This study |
| YA-6 | YA-5, F1::P_TEF_*-CrMAS*^L323A^*-*T_XPR2_ | This study |
| YA-7 | YA-6, B4::P_TEF_*-CrMAS*^L323A^*-*T_XPR2_ | This study |
| YA-8 | YA-5, E5-P_TEF_-*CrMAS*^L323A^-T_XPR2_ | This study |
| YA-9 | YA-8, B3-P_TEF_-*ZWF1*-T_XPR2_-P_TDH_-*ZWF1*-T_ICLt_ | This study |
| YA-10 | YA-9, F4-P_TEF_-*GND1*-T_XPR2_ | This study |

**Table S4. *ERG1/SQLE* protein sequences included in the multiple sequence alignment**

| **Kingdom** | **Organism** | **Accession (UniProt / GeneID)** | **Protein length (aa)** |
| --- | --- | --- | --- |
| Fungi | *Yarrowia lipolytica* | Q6C5R8 | 489 |
| Fungi | *Saccharomyces cerevisiae* | P32476 | 496 |
| Fungi | *Candida albicans* | Q92206 | 520 |
| Fungi | *Aspergillus fumigatus* | E9R5G2 | 537 |
| Mammalia | *Homo sapiens* | Q14534 | 574 |
| Mammalia | *Mus musculus* | P52019 | 572 |
| Fungi | *Cordyceps militaris* | G3JM40 | 585 |
| Fungi | *Ophidiomyces ophidiicola* | GeneID: 73307128 | 494 |
| Fungi | *Aspergillus flavus* | A0A5N6GGK7 | 388 |
| Fungi | *Coemansia asiatica* | A0A9W8CJR1 | 544 |
| Fungi | *Fusarium falciforme* | A0A9W8R7T5 | 498 |
| Fungi | *Didymella glomerata* | A0A9W8X0E1 | 512 |
| Fungi | *Exophiala dermatitidis* | H6BYC1 | 508 |
| Fungi | *Nakaseomyces glabratus* | O13306 | 489 |
| Fungi | *Brettanomyces bruxellensis* | A0A7D9GYI5 | 495 |
| Fungi | *Colletotrichum fructicola* | A0A7J6JD06 | 496 |
| Fungi | *Candida jiufengensis* | GeneID: 76163671 | 478 |

**Table S4. Codon-optimized nucleotide sequence of the heterologous gene used in this study.**

***CrMAS (Catharanthus rosеus)* Sequence (5'-3')**

ATGTGGAAGCTCAAGATTGCCAAGGGTAAGGGGCCTTACCTATACAGCACCAACAACTTCGTGGGTCGACAAATTTGGGAATACGATCCCAACGCAGGAACTCCCCAAGAGCGAGAGGCCTTTGAGAAGGCCCGCGAACAGTTCCGAAACAACAGAAAGAAGGGGGTGCACAATCCCTGTGCAGATCTGTTCATGAGAATGCAGCTGATAAAAGAGAACGGTATCGACCTAATGTCCATTCCGCCTGTGCGAGTCGAGGAGAAGGAGGAGCTCACGTTCGAGAAGACCACCATTGCTGTCAAGAAGGCCCTCCGGCTCAACCGTGCCATTCAGGCCACGGACGGCCACTGGCCTGCTGAGAATGCTGGTCCAATGTTCTTCACTCCTCCACTTCTCATTGCGCTGTACATTAGTGGAGCCATCAACACCATTCTGACTTCGGAACACAAGAAAGAGCTGGTCCGCTACATCTACAACCACCAGAACGAGGATGGCGGCTGGGGTTTTTACATTGAGGGACATTCTACTATGATTGGCAGCGCGCTGAGTTACGTGGCTCTCCGGCTATTAGGCGAAGGTCCCGATGATGGAGATGGTGCTGTAGGTAGAGGACGTCAGTGGATCCTCGACCACGGAGGAGCCACGGGTATCCCCTCTTGGGGTAAGACCTACCTTTCTGTTCTCGGTGTCTACGACTGGGATGGATGTAACCCGCTGCCGCCCGAATTCTGGCTCTTTCCTTCGTTCTTCCCTTATCATCCCGCTAAGATGTGGTGCTACTGCCGGACAACATACATGCCGATGTCTTACCTGTACGGAAAGAAGTATCACGGACCTCTGACCCATCTCGTTATGCAACTCAGGCAGGAGATCCACGTGAAGCCCTATGATCAGATTGACTGGAACAAGGCACGACACGATTGCTGCAAGGACGATCTGTACTACCCCCATTCATTCATCCAGGATGCCCTGTGGGACACTCTTAACTACTTTTCAGAGCCCGTCATGAGACGATGGCCCTGTAACAAGATTAGAGAAAAGGCCATGCGAAAGTGCATCAAATATATGCGATACGGCGCCGAGGAGTCGCGCTATATCACCATTGGCTGTGTGGAGAAATCGCTGCAAATGATGTGCTGGTGTGCCCACGACCCGAACTGCGACGAGTTCAAATACCACCTTGCACGTGTGCCTGACTACCTCTGGCTGGCAGAAGATGGCATGAAGATGCAGAGCTTTGGATCTCAGCTCTGGGACTGTACCCTAGCTACCCAGGCTATCATAGCAACCGGAATGGTCGAGGAATATGGAGACACAATCAAAAAGGCGCACTTCTACATCAAGGAGAGCCAGGTGAAGGAGAACCCAAAGGAGGACTTCAAGGCCATGTACCGACATTTCACTAAGGGGTCCTGGACATTTTCCGACCAGGACCAGGGCTGGGTGGTCTCGGACTGCACCGCCGAAGCTCTGAAGTGTCTTCTCGTCTGTTCTCAAATGCCACAAGACCTTGCTGGTGAAAAAGCGGACGTGGAGCGGTTATACGACGCCGTCAACGTCCTTTTGTATCTTCAGTCTCCCGAGTCTGGAGGATTTGCTATCTGGGAGCCACCTGTTCCTCAGCCCTATCTGCAAGTTCTGAACCCCTCTGAATTGTTTGCCGACATCGTTGTCGAACAGGAGCATGTTGAAAATACTGCCTCCATCGTTCAGGCGCTTGTACTGTTTAAACGATTGCACCCCGGCCATCGGGAGAAGGAGATTGAAGTGTCGGTTTCCAAGGCTGTGCGTTTTCTGGAGGGCCGACAGTGGCCCGATGGATCCTGGTACGGGTACTGGGGCATCTGCTTCCTGTACGGCACCATGTTTGTGCTCGGTGGGCTGACTGCGGCTGGCAAGACTTACAAGAACTCCGAGGCTATTCGAAAAGCCGTCAAGTTCTATTTATCAACGCAAAATGAGGAGGGAGGATGGGGAGAGTGTCTGGAATCGTGTCCCTCCATGAAGTACATTCCTCTTGAGGGCAATCGAACCAATCTGGTGCAGACATCCTGGGCTATGCTAGGCCTGATGTACGGTGGCCAGGCAGAACGTGACCCCACCCCTCTCCACAAAGCTGCCAAGCTGCTGATCAACGCCCAGATGGACGACGGCGATTTTCCTCAGCAGGAGATCACTGGTGTATACATGAAGAACTGCATGTTGCATTACGCCGAGTACCGGAACATTTTCCCCCTGTGGGCCTTGGCGGAGTACCGCAAGCGAGTGTGGCCTACGAAAGCTTTGTGA
